# The Path Planning of Mobile Robot by Neural Networks and Hierarchical Reinforcement Learning

**DOI:** 10.3389/fnbot.2020.00063

**Published:** 2020-10-02

**Authors:** Jinglun Yu, Yuancheng Su, Yifan Liao

**Affiliations:** Chongqing University-University of Cincinnati Joint Co-op Institute, Chongqing University, Chongqing, China

**Keywords:** neural network, hierarchical reinforcement learning, mobile robot, path planning, fusion algorithm

## Abstract

Existing mobile robots cannot complete some functions. To solve these problems, which include autonomous learning in path planning, the slow convergence of path planning, and planned paths that are not smooth, it is possible to utilize neural networks to enable to the robot to perceive the environment and perform feature extraction, which enables them to have a fitness of environment to state action function. By mapping the current state of these actions through Hierarchical Reinforcement Learning (HRL), the needs of mobile robots are met. It is possible to construct a path planning model for mobile robots based on neural networks and HRL. In this article, the proposed algorithm is compared with different algorithms in path planning. It underwent a performance evaluation to obtain an optimal learning algorithm system. The optimal algorithm system was tested in different environments and scenarios to obtain optimal learning conditions, thereby verifying the effectiveness of the proposed algorithm. Deep Deterministic Policy Gradient (DDPG), a path planning algorithm for mobile robots based on neural networks and hierarchical reinforcement learning, performed better in all aspects than other algorithms. Specifically, when compared with Double Deep Q-Learning (DDQN), DDPG has a shorter path planning time and a reduced number of path steps. When introducing an influence value, this algorithm shortens the convergence time by 91% compared with the Q-learning algorithm and improves the smoothness of the planned path by 79%. The algorithm has a good generalization effect in different scenarios. These results have significance for research on guiding, the precise positioning, and path planning of mobile robots.

## Introduction

Mobile robot autonomous navigation can be divided into three subsystems: information perception, behavior decision-making, and manipulation control. Path planning is the basis of mobile robot navigation and control (Ghosh et al., 2017; Orozco-Rosas et al., 2019). The goal of mobile robot path planning is to find a path from the current position to the target position. The path should be as short as possible, the smoothness of the path should meet the dynamics of the mobile robot, and the safety of the path should be collision-free (Han and Seo, 2017).

Depending on how much information is known about the environment in the path planning process, path planning can be divided into global path planning and local path planning (Li and Chou, 2018). There are many methods of path planning. According to specific algorithms and strategies, path planning algorithms can be roughly divided into four types: template matching, artificial potential field, map construction, and artificial intelligence (Zhao et al., 2018). Each type of path planning algorithm has an optimal application scenario and limitations. The current path planning of mobile robots relies heavily on the surrounding environment. In addition to the limitations of traditional path planning, robots cannot complete their learning and judgment in complex environments, a bottleneck in the development of research in this field (Bakdi et al., 2017). It is therefore particularly important to develop a path planning method with low reliance on the environment, which can quickly adapt to the surrounding environment.

The Deep Q-Learning Network (DQN) is a way of modeling the environment and calculating the collision energy function, which is the main cause of a loss in functionality (Ohnishi et al., 2019). To realize the path planning process, the neural network is trained to minimize the loss function through the gradient descent method. To enable better generalization ability in the neural network, various sample data are needed for learning and training, however, an over large data sample will increase the training time (Shen et al., 2019a; Sung et al., 2020).

Deep Reinforcement Learning (DRL), as an important machine learning method, has received more attention and there are increasing applications of it in robot path planning DRL (Arulkumaran et al., 2017). The agent obtains knowledge through the exploration of an environment and learns using a process of trial and error. The DRL method has obvious advantages in path planning and requires less prior information about the environment (Wulfmeier et al., 2017; Zheng and Liu, 2020).

Unlike the supervised learning method, reinforcement learning does not require much sample data for training, like neural network methods, and acquires sample data during the training process. In recent years, scholars have focused on using new algorithms or fusion algorithms to improve the performance of mobile robots (Yan and Xu, 2018). Lei et al. found that adding the Q-Learning algorithm to the reinforcement learning path enhances the ability of robots to dynamically avoid obstacles and local planning in the environment (Lei et al., 2018; Liu et al., 2019). Wang et al. found that compared with Distributed DQN (DDQN) algorithm, the Tree Double Deep Network (TDDQN) has the advantages of fast convergence speed and low loss (Wang P. et al., 2020). By using a neural network to strengthen the learning path planning system, Wen et al. suggested that the mobile robot can be navigated to a target position without colliding with any obstacles and other mobile robots, and this method was successfully applied to the physical robot platform (Wen et al., 2020). Botteghi et al. introduced a reward function training strategy in the fusion algorithm, which not only outperformed the standard reward function in terms of convergence speed but also reduced the number of collisions by 36.9% of iteration steps (Shen et al., 2019b; Botteghi et al., 2020). Therefore, the fusion algorithm has obvious advantages in path planning and algorithm performance. However, the path planning performance of current fusion algorithms is not outstanding.

Taking into account the shortcomings of these research results, we designed a mobile robot path planning system based on neural networks and hierarchical reinforcement learning. Through neural networks, this system perceives the environment and performs feature extraction to realize the fitting from the environment to the state action function (Chen, 2018). The mapping of the current state to the action of the hierarchical reinforcement learning is satisfied through the enhancement function, thereby realizing the demand for mobile robots. Theoretically, the organic combination of the two can improve the performance of mobile robots in path planning. Therefore, in this study, the algorithm was embedded into a mobile robot, and the designed algorithm was verified by comparing it with other path planning algorithms in different environments and scenarios. The initial *Q*-value of the proposed algorithm sped up the convergence speed, redefined the number of states, as well as the direction of motion, and step length. The real-time performance of the mobile robot's path planning and smoothness was significantly improved, and could be used to guide robot movement, and improve algorithm mobility (Liu and Wang, 2019).

## Methods

### Mobile Robot Path Planning Model

**Figure 1:**
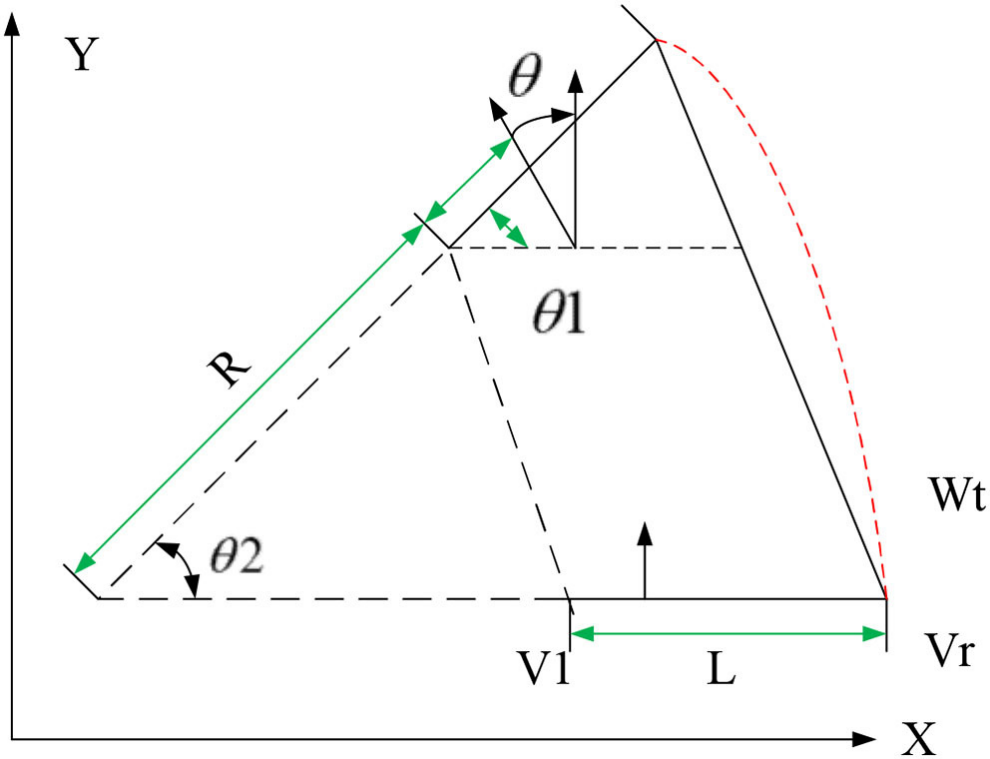
The path planning motion model of mobile robots.

The path planning task explored in this study is based on a two-wheel differential mobile robot. The robot can control the speed of its two driving wheels to achieve arbitrary trajectory movements such as linear movement, turning, and turning around in circles. Figure 1 shows the pose of the robot at adjacent time intervals, based on which kinematic model is established.

The world coordinate system pose of the mobile robot at time t is set to $Wt={\left[x_{t},y_{t},\theta_{t}\right]}^{T}$; if the world coordinate pose of the mobile robot at time *t* + Δ*t* is $W_{t+\Delta \text{t}}={\left[x_{t+\Delta \text{t}},y_{t+\Delta \text{t}},\theta_{t+\Delta \text{t}}\right]}^{T}$, the distance between the left and right driving wheels is L, the speeds of the left and right driving wheels are *v*_*l*_ and [[Mathtype-mtef1-eqn-5.mtf]], and the robot linear speed and angular speed are respectively *v* and ω, the speed *v* of the mobile robot in the ideal motion state is:

(1) $$\begin{array}{l} v=\frac{v_{l}+v_{r}}{2} \end{array}$$

The angular velocity of the robot is:

(2) $$\begin{array}{l} \omega =\frac{v_{l}-v_{r}}{L} \end{array}$$

The instantaneous curvature radius R is:

(3) $$\begin{array}{l} R=\frac{v}{\omega } \end{array}$$

As shown in Figure 1, θ_1_ = θ_2_ = θ, after Δ*t*, the heading angle of the robot changes as follows:

(4) $$\begin{array}{l} \theta_{\text{t}+\Delta \text{t}}=\theta_{t}+\theta \end{array}$$

The motion from position $Wt={\left[x_{t},y_{t},\theta_{t}\right]}^{T}$ to $W_{t+\Delta \text{t}}={\left[x_{t+\Delta \text{t}},y_{t+\Delta \text{t}},\theta_{t+\Delta \text{t}}\right]}^{T}$ can be regarded as a circular arc with radius R. If the arc is used to approximate the actual trajectory of the robot, the geometric relationship should be:

(5) $$\begin{array}{l} \left[\begin{array}{l} x_{t+\Delta t}\\ y_{t+\Delta t}\\ \theta_{t+\Delta t} \end{array}\right]=\left[\begin{array}{l} x_{t}+R\left(\text{sin}\left(\theta_{t}+\theta \right)-\text{sin}\theta_{t}\right)\\ y_{t}+R\left(\text{cos}\left(\theta_{t}+\theta \right)-\text{cos}\theta_{t}\right)\\ \theta_{t}+\theta \end{array}\right],\theta \neq 0 \end{array}$$

Combining the above equations, the motion equation of the differential mobile robot can be obtained as:

(6) $$\begin{array}{l} \left[\begin{array}{l} x_{t+\Delta t}\\ y_{t+\Delta t}\\ \theta_{t+\Delta t} \end{array}\right]=\left[\begin{array}{l} x_{t}+\frac{L\left(v_{r}+v_{l}\right)}{2\left(v_{r}-v_{l}\right)}(R\left(\text{sin}\left(\theta_{t}+\theta \right)-\text{sin}\theta_{t}\right)\\ y_{t}+\frac{L\left(v_{r}+v_{l}\right)}{2\left(v_{r}-v_{l}\right)}R\left(\text{cos}\left(\theta_{t}+\theta \right)-\text{cos}\theta_{t}\right)\\ \theta_{t}+\theta \end{array}\right],\theta \neq 0 \end{array}$$

### ANN

ANN is a mathematical or computational model that simulates the structure and function of biological neural networks, which is used to estimate or approximate functions. With the continuous deepening of research works on ANNs, it has made great breakthroughs in the fields of speech recognition, pattern recognition, automatic control, and predictive estimation. ANN has successfully solved many problems that are difficult for computers to solve, showing good performance.

In the practical application of ANN, most neural network models use a backpropagation neural network (BPNN) and its transformations, which have good nonlinear mapping ability, self-learning ability, and fault tolerance. It mainly uses many aspects such as pattern recognition, function approximation, data compression, prediction estimation, and classification. Therefore, the most representative BPNN is chosen as the basis of modeling to analyze the robot path. An ANN is usually composed of multiple BPNN layers and multiple neurons, which are mainly divided into an input layer, a hidden layer, and an output layer, where the input vector should be:

(7) $$\begin{array}{l} x=\left[x_{1},x_{2},x_{3}...x_{i},...x_{m}\right],i=1,2,....m \end{array}$$

The output vector should be:

(8) $$\begin{array}{l} y=\left[y_{1},y_{2},y_{3}...y_{\text{k}},...y_{n}\right],\text{k}=1,2,....n \end{array}$$

The neuron input of the hidden layer should be:

(9) $$\begin{array}{l} {\text{h}}^{\left(\text{l}\right)}=\left[{{\text{h}}^{\left(\text{l}\right)}}_{1},{{\text{h}}^{\left(\text{l}\right)}}_{2},{{\text{h}}^{\left(\text{l}\right)}}_{3}...{{\text{h}}^{\left(\text{l}\right)}}_{j},...{{\text{h}}^{\left(\text{l}\right)}}_{sl}\right],j=1,2,....sl \end{array}$$

Where: sl is the number of neurons in layer 1; assuming that ${{\text{w}}^{\left(\text{l}\right)}}_{\text{ij}}$ is the connection weight between the j-th neuron in layer 1-1, ${{\text{b}}^{\left(\text{l}\right)}}_{\text{i}}$ is the threshold of the i-th neuron in layer 1, and $ne{t^{\left(l\right)}}_{i}$ is the input of the i-th neuron in layer 1, then the following equation is obtained:

(10) $$\begin{array}{l} {{\text{h}}^{\left(\text{l}\right)}}_{\text{i}}=f\left(ne{t^{\left(l\right)}}_{i}\right) \end{array}$$

(11) $$\begin{array}{l} ne{t^{\left(l\right)}}_{i}=\overset{sl-1}{\underset{j=1}{\sum }}{w^{\left(l\right)}}_{ij}{{\text{h}}^{\left(l-1\right)}}_{\text{j}}+{{\text{b}}^{\left(l\right)}}_{i} \end{array}$$

**Figure 2:**
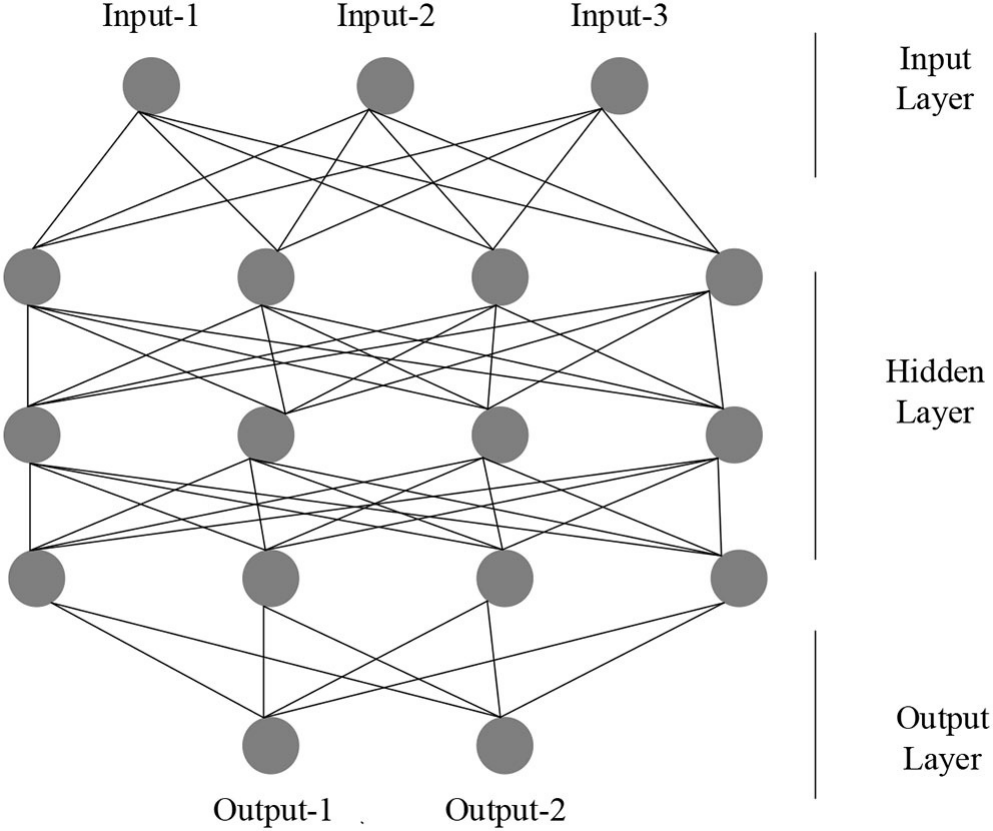
Structure of ANN.

Here, the functions of the input layer to the output layer use the S-type corresponding TANSIG function, the output layer uses the PURELIN linear function, the learning rules use the TRINGDX function, and the performance evaluation uses the MES function, where the model number is set to 1,000 times and the accuracy is set to 0.0001. The rest are the default parameters of the system, and the specific structure is shown in Figure 2.

### Reinforcement Learning

**Figure 3:**
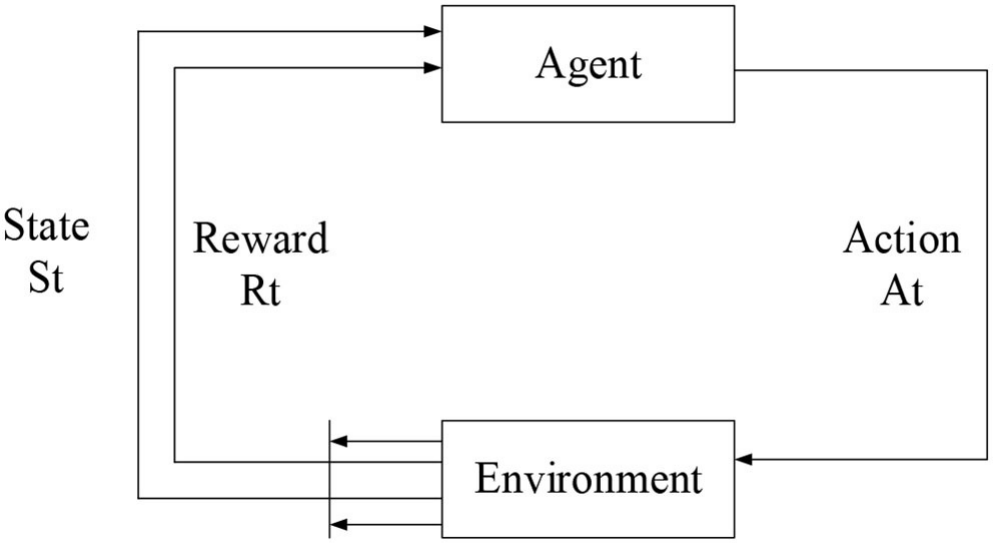
The schematic diagram of the reinforcement learning model.

Reinforcement learning is a machine learning method that learns by interacting with the environment. An Agent uses reinforcement learning methods to learn, which is to acquire knowledge from a sequence of actions obtained by exploration. Its sample data is not existing, meaning it is different from the supervised learning process. After an Agent executes an action, it will get feedback from the environment. This feedback is the evaluation of the action made by the environment and is a process of “trial and error.” The evaluation of the action made by the environment is the immediate reward value received by the Agent. The immediate reward is an enhanced signal, which indicates the impact of the execution of this action on the result. The larger the value is, the better the effect is, otherwise it will have a poor impact. The reinforcement learning model is shown in Figure 3. The learning process of the reinforcement learning method is a heuristic process. It continuously tries through random units, searches for the optimal action to obtain the enhanced signal of the environment, and increases the probability that the optimal action is selected by the iterative update, thereby finding a set of optimal solutions (a set of action sequences with the highest reward value).

The reinforcement signal in reinforcement learning comes from the immediate reward of environmental feedback. This reward value indicates the quality of the action performed instead of telling the machine what the correct action is. The process of the machine interacting with the environment can be regarded as a Markov Decision Processing (MDP). As long as the random variable set {*X*_1_, *X*_2_, *X*_3_....*X*_t_} satisfies the following equation, the set will have Markov attributes:

(12) $$\begin{array}{l} P_{r}(X_{t+1}=x|X_{t}=x_{t},X_{t-1}=x_{t-1},...X_{1}=x_{1})\\ \;=P_{r}\left(X_{t+1}=x|X_{t}=x_{t}\right) \end{array}$$

Once the state x is determined, the actions before the state are not correlated to the actions after the state and are independent of each other. Among them, the state set S, the action set A, the reward function R, the state transition function T, and the objective function constitute the MDP. The state process of the transition is as follows:

(13) $$\begin{array}{l} T\left(s,\alpha ,s^{\prime }\right)=P_{r}\left(s_{t+1}=s^{\prime }|s_{t}=s,\alpha_{t}=a\right) \end{array}$$

The process of Markov's decision is mainly to realize a set of action sequences α = π(s) so that the cumulative discount reward $\overset{\infty }{\underset{t=0}{\sum }}\gamma^{\prime }R\left(s_{t},a_{t}\right)$ reaches the maximum value. Through the iteration of values, the optimization problem of MDP can be realized. The function of the optimal value is defined as:

(14) $$\begin{array}{l} V*\left(s\right)=\text{max}\left(\text{R}\left(\text{s},\alpha \right)+\gamma \underset{{\text{s}}^{\prime }\in \text{S}}{\sum }\text{T}\left(\text{s},\text{a},{\text{s}}^{\prime }\right)\text{V}*\left({\text{s}}^{\prime }\right)\right),\forall \text{s}\in \text{S} \end{array}$$

Then, the optimal strategy is calculated as follows:

(15) $$\begin{array}{l} \pi \left(\text{s}\right)=\text{arg}|\max\left(\text{R}\left(\text{s},\alpha \right)+\gamma \underset{{\text{s}}^{\prime }\in \text{S}}{\sum }\text{T}\left(\text{s},\text{a},{\text{s}}^{\prime }\right)\text{V}*\left({\text{s}}^{\prime }\right)\right) \end{array}$$

The reinforcement learning system is mainly composed of three parts: reward function, value function, and action selection strategy. Among them, reinforcement function is divided into continuous reward function. By establishing a mathematical model between the state and environmental feedback perceived by the Agent at each moment, the Agent can obtain the evaluation of the environment in each state, giving more guidance information during the Agent training process, and the Agent can find the optimal strategy faster. The calculation is as follows:

(16) $$\begin{array}{l} R_{t}=f\left(s_{t},i_{t}\right) \end{array}$$

The discrete reward functions require less a-priori information and are simple to construct, which have better applications in exploration and learning in unknown environments. The calculation is as follows:

(17) $$\begin{array}{l} R_{t}=\{\begin{array}{l} 1\text{ Perform optimal actions}\\ -1\text{ Perform the worst action}\\ 0\text{ Other situations} \end{array} \end{array}$$

The reward function only gives the reward of the currently executed action, but this does not guarantee that each action can get a reward. As the training progresses, the value function continuously optimizes and converges, and the action is selected by strategy in a state, which ensures that each action will get not only the largest reward but also the largest cumulative discount reward, of which the limited non-discount cumulative reward function is:

(18) $$\begin{array}{l} V^{\pi }\left(s_{t}\right)=\overset{h}{\underset{t=0}{\sum }}r_{t} \end{array}$$

Where: *r*_*t*_ is the reward immediately obtained by the machine at time t, and the cumulative reward is the accumulation of the immediate rewards obtained from the starting state to the target state. The unlimited discount reward function is:

(19) $$\begin{array}{l} V^{\pi }\left(s_{t}\right)=\overset{h}{\underset{t=0}{\sum }}\gamma^{\prime }r_{t+1}0\leq \gamma \leq 1 \end{array}$$

Where: γ′ is the discount factor, and the value range is 0 ≤ γ ≤ 1, which represents the limit of reinforcement learning. The value function pays more attention to future rewards. The average reward function is:

(20) $$\begin{array}{l} V^{\pi }\left(s_{t}\right)=\underset{h\to \infty }{\lim}\left(\frac{1}{h}\overset{h}{\underset{t=0}{\sum }}r_{t}\right) \end{array}$$

After learning, the optimal strategy can use the value function obtained by training to select the action strategy. The equation is as follows:

(21) $$\begin{array}{l} \pi *=\text{arg max }{\text{V}}^{\pi }\left(\text{s}\right),\forall \text{s}\in \text{S} \end{array}$$

The action selection strategy of Softmax is used to analyze the probability of the action, which is generally described by the Boltzmann distribution function. The mathematical model is as follows, where T is the temperature control coefficient.

(22) $$\begin{array}{l} p\left(at/s\right)=)=\frac{k^{Vi/T}}{\underset{\alpha \in A}{\sum }k^{Vi/T}} \end{array}$$

### Different Path Planning Recognition Algorithms

Here, different algorithms are compared to determine the advantages of the proposed algorithm. There are many recognition algorithms for the path planning of mobile robots. These path planning algorithms are all based on the principle of feature point positioning, which changes in any direction of the images mainly through a Gaussian window. Through this movement, the correlation matrix of different windows is calculated and the image data of the environment are obtained.

**Figure 4:**
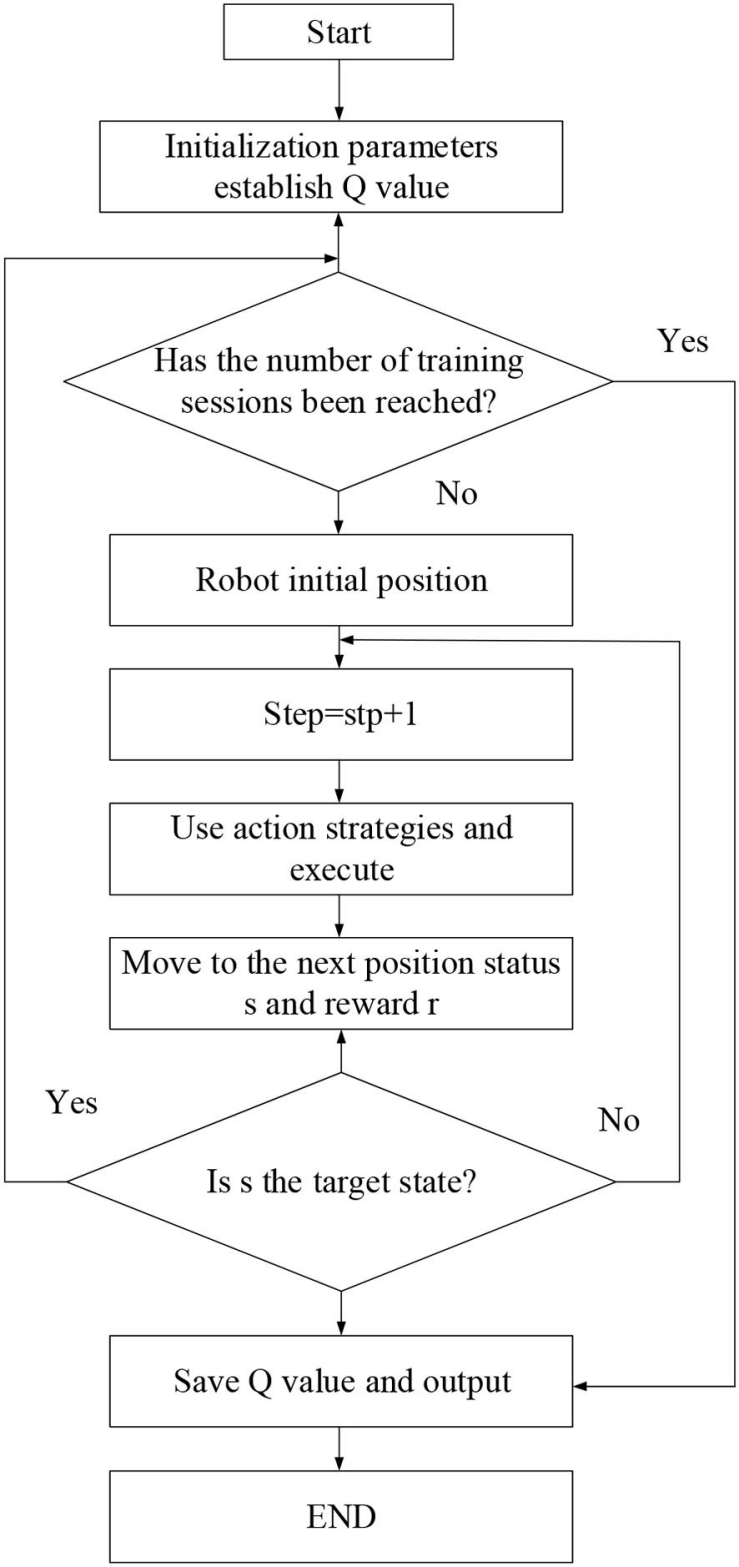
Schematic diagram of Q-Learning path planning method.

**Figure 5:**
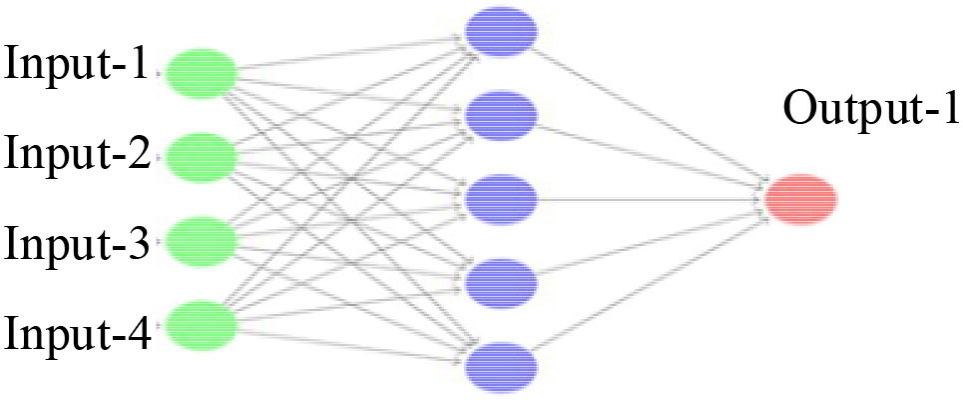
Schematic diagram of the neural network approximation function structure.

**Figure 6:**
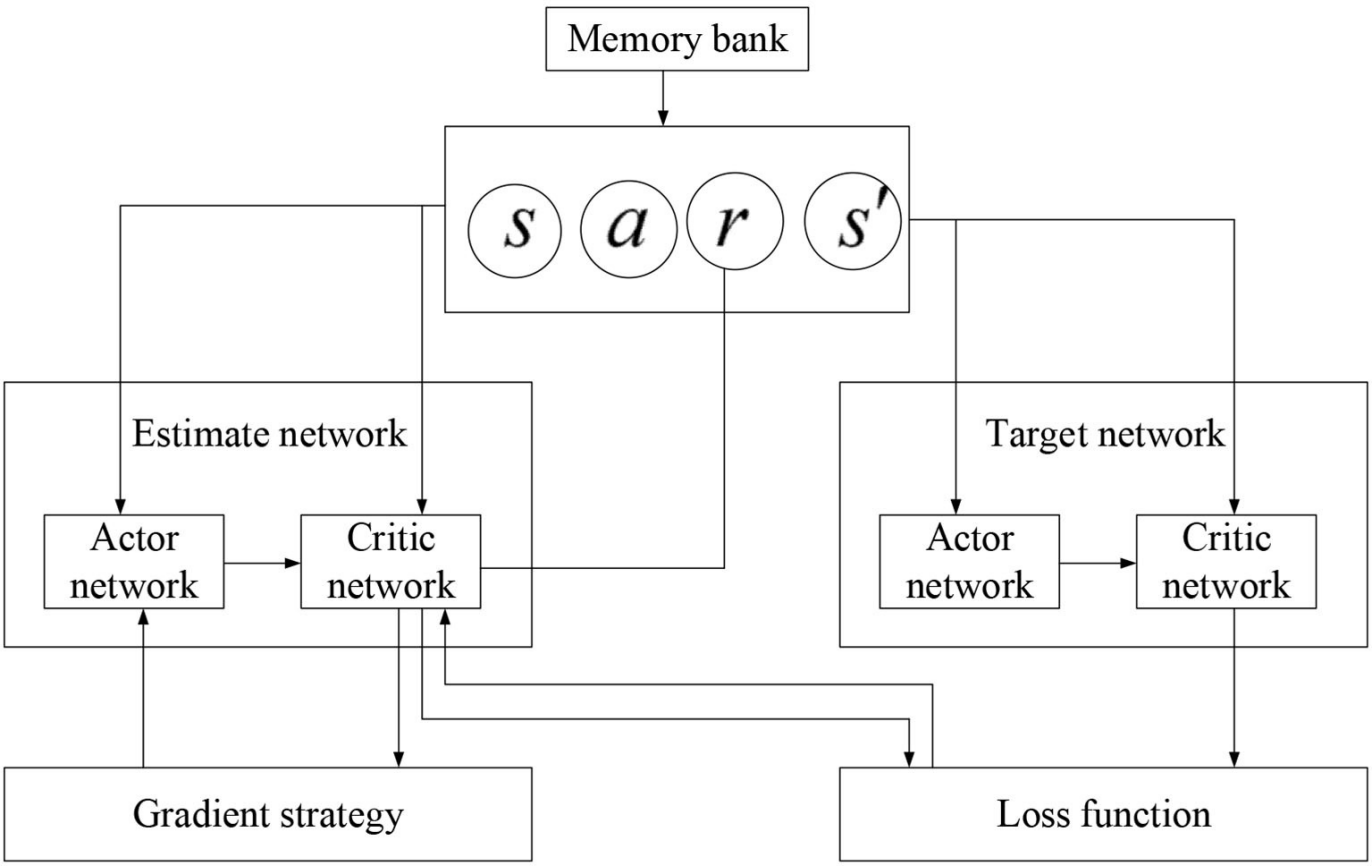
Structure of DDPG network algorithm.

1. The Q-Learning algorithm is a table-valued learning algorithm because the state-action Q value table is established during the interaction between the machine and the environment. The reward in the environment will affect the *Q*-value corresponding to the state-action. The *Q*-value of the correct behavior is gradually increased under the positive reward, and the *Q*-value corresponding to the wrong behavior will also be reduced under the negative reward. The optimal action is selected in the action selection strategy to make the Agent obtain the optimal behavior strategy (Wei et al., 2016; Zhu et al., 2017). The method of updating the *Q*-value is as follows in Figure 4:
2. The DQN algorithm is a process of using the neural network to approximate the value function. As shown in Figure 5, the optimal value function *Q*(*s*, α, θ) is approximated by adjusting the weight of the neural network. The update value function changes the parameters. After the neural network training is completed, the parameters are determined, and the corresponding function value will not change anymore. The training process then converges (Liu and Hodgins, 2017; Zhu et al., 2017). The location update equation is:
  (23) $$\begin{array}{l} \theta_{t+1}=\theta_{t}+\alpha [r+\gamma \text{ max }Q\left(s,\alpha ,\theta \right]\Delta Q\left(s,\alpha ,\theta \right) \end{array}$$
3. The Potential DQN (PDQN) algorithm is an improvement to the DQN algorithm. Its major purpose is to accelerate the running speed of the algorithm. On this basis, the artificial potential field method is added (Gupta et al., 2019). The gravitational field is calculated as follows:
  (24) $$\begin{array}{l} U\left(X\right)=\frac{1}{2}k{\left(X-X_{g}\right)}^{2} \end{array}$$
  Where: k is the gain coefficient, X is the current position of the mobile robot, Xg is the target position, j is the planning adjustment reward, and the relationship between reward and gravity is as follows:
  (25) $$\begin{array}{l} r=jU\left(X\right) \end{array}$$
4. Actor-Critic (A3C) algorithm is a way of reinforcement learning. It introduces an evaluation mechanism to solve the high variance problem. It utilizes a neural network to predict the selected action and directly passes the prediction result back to increase the probability that the action is selected next time. If the reward function shows that the selected action is not optimal, the probability that the action is selected next time will be reduced (Haarnoja et al., 2018). The strategy gradient equation is as follows:
  (26) $$\begin{array}{l} \Delta_{\theta }J\theta =\frac{1}{T}\overset{T}{\underset{t}{\sum }}\Delta_{\theta }\log\pi \left(\alpha_{t}|s_{t};\theta \right)(\overset{n}{\underset{i=1}{\sum }}\gamma^{i-1}rt+1+v\left(s_{t}+n\right)\\ \;-v\left(s_{t}\right))+\beta \Delta_{\theta }H(\pi \left(s_{t};\theta \right) \end{array}$$
5. The Deep Deterministic Policy Gradient (DDPG) algorithm is an algorithm with a lot of improvements to DQN, in which the A3C algorithm is added. It is a fusion algorithm of neural network and reinforcement learning. The specific improvement details are shown in Figure 6.
6. THE double DQN (DDQN) algorithm estimates the maximum action in the target network through the network and uses this estimated action to select *Q*(*s*) in the target network (Zhang et al., 2018; Han et al., 2019). Then, the goals of TD should be:
  (27) $$\begin{array}{l} {Y_{\text{t}}}^{D\text{ouble}DQN}=R_{t+1}+\gamma Q\left(s_{t+1},\arg\max Q\left(s_{t+1},{\theta_{t}}^{\prime }\right)\right) \end{array}$$

### Construction and Monitoring of Simulation Environment

The simulation environment mainly uses the multimedia framework pyglet under Python to design the interactive applications as the simulation platform. A 200^*^200-pixel static environment is built in the experiment. In the environment, the mobile robot is no longer a particle but is represented by a blue circle of 10^*^10 size. The green circle S represents the starting coordinate. The pixel coordinate of the starting position is (5, 5). The purple circle represents the target position and the five black areas of different sizes in the figure are the positions of obstacles. The white area indicates that there are no obstacles in the map, in which the robot can move freely. Since the robot has size in the real world when the boundary of the mobile robot is in contact with the boundary of the obstacle area, it is considered to have collided, and after the collision, it is considered to have failed and is returned to the starting position. The state of the experiment is represented by the rasterized state.

The detection mainly uses the summary.value.add () function in TensorFlow to add variables to the monitoring log. The changes in training process data can be viewed through TensorFlow. After learning, the neural network parameters are saved by using the tf.train.Saver () function and the neural network is reloaded and run again to indicate the effect after the learning is completed. The experimental results show that the mobile robot can avoid dynamic obstacles in time and find an optimal path to reach the target position after avoiding the dynamic obstacles. In the experiment, the copy network value function, the average number of steps used to reach the target position, and the average cumulative reward of the copy network are saved. At the end of the learning and training process, the changing process of the three data can be viewed through TensorBoard.

## Results and Discussions

### Experimental Results of Different Path Planning Algorithms of Mobile Robot

**Figure 7:**
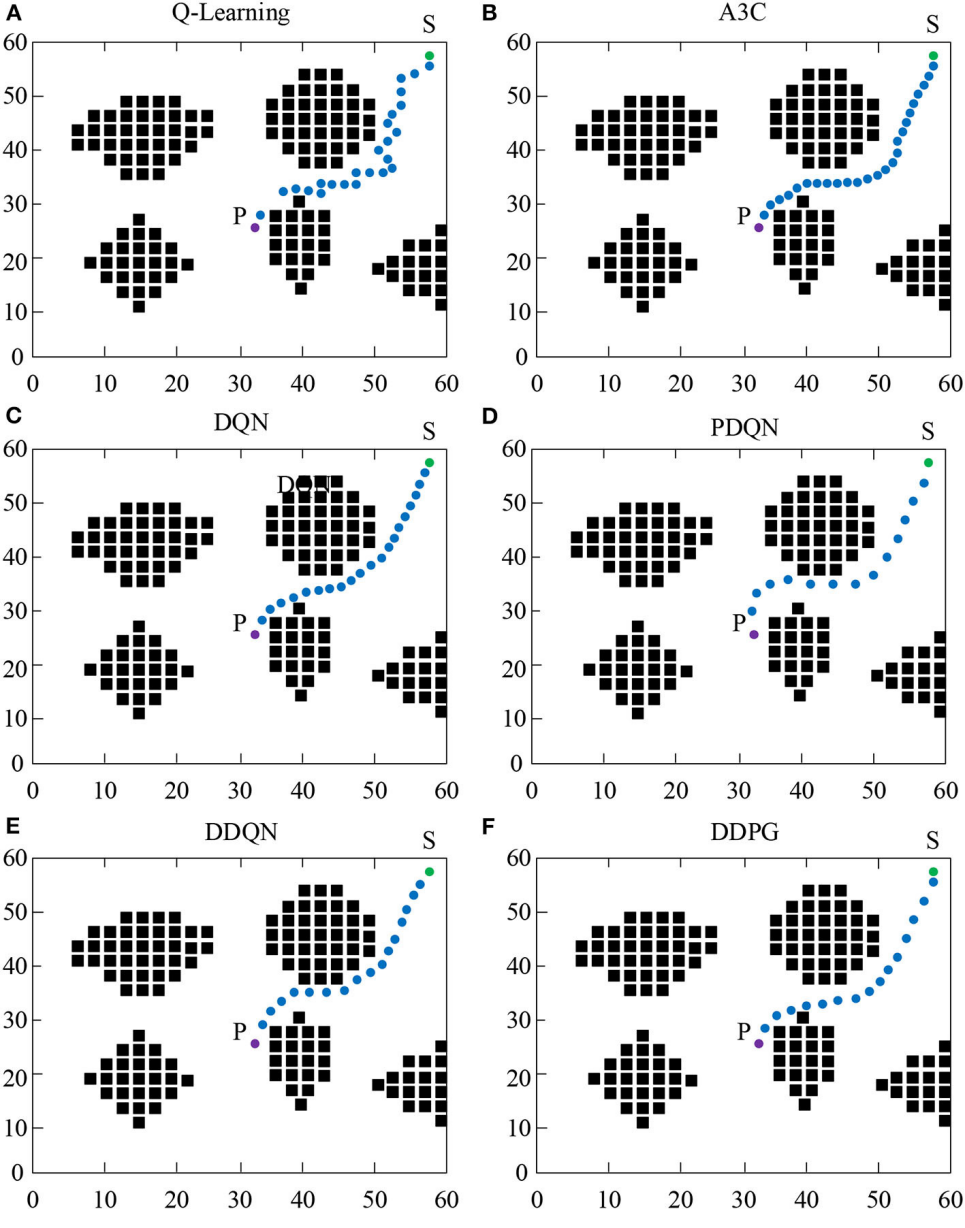
Experimental results of different path planning algorithms of mobile robot.

Figure 7 shows the experimental results of the path planning of mobile robot under different algorithms. As shown in Figure 7, under the same starting and ending conditions, all algorithms can effectively avoid obstacles. Comparing Figures 7A,B, it was found that in the traditional Q-Learning and A3C algorithms, the reinforcement learning algorithm effectively reduces the number of path steps. Comparing Figures 7A,C, it was found that the introduction of a neural network algorithm based on the traditional Q-Learning algorithm can greatly reduce the number of paths and achieve the same effect as the reinforcement learning algorithm. Comparing Figures 7C,D, it was found that the introduction of the force field based on the neural network has greatly accelerated the running speed of the algorithm, causing a significant reduction in the number of steps. Although the algorithm can effectively avoid obstacles, it has taken many useless paths. Therefore, the DDQN algorithm of Q value accumulation was added. As shown in Figure 7E, the algorithm can effectively utilize the neural network to learn and achieve the minimum number of steps. Compared to the DQN algorithm, the running speed of DDQN was improved and compared to the PDQN algorithm, the DDQN can find the optimal path. As shown in Figure 7F, a reinforcement learning algorithm was added based on the neural network. It was found that compared to the DDQN algorithm, it runs faster and has an optimal path. According to the above results, the fusion algorithm using a neural network and reinforcement learning has better performance in the path experiment.

### Performance Evaluation of Different Path Planning Algorithms of Mobile Robot

**Figure 8:**
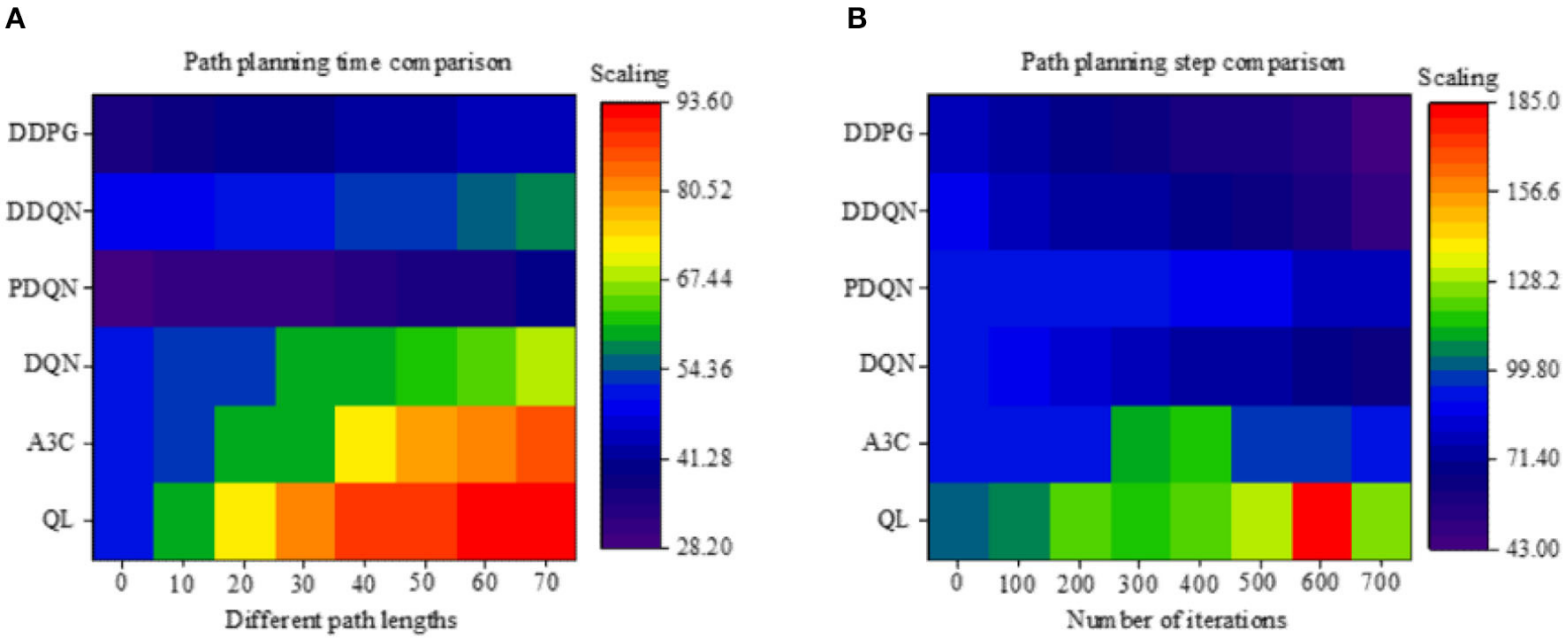
Performance evaluation of time and steps of different mobile robot path planning algorithms (QL algorithm represents the Q-Learning algorithm).

Figure 8A illustrates the path planning time of different algorithms under different path lengths. The results show that as the path length increases, the path planning time is also increasing, where the time required is proportional to the path length. As far as different algorithms are concerned, the traditional Q-Learning algorithm takes the longest time, with an average of 78.35 s. The PDQN takes the shortest time because the algorithm introduces a force field, causing the algorithm to be improved continuously. The DDPG algorithm based on neural networks and HRL marks the second position, which takes an average of 40.7 s and is 48.05% higher than the traditional algorithm, 31.01% higher than the DQN algorithm of the neural network, and 40.1% higher than the reinforcement algorithm.

Figure 8B illustrates the number of path steps of different algorithms at different iteration times. As the number of iterations increases, it does not affect the Q-Learning and A3C algorithms because these two algorithms do not have deep learning capabilities. With the increase in the number of iterations, in terms of other algorithms, the number of path steps continues to decrease under the same path. Of the different algorithms, the reinforcement learning algorithm is significantly better than the traditional Q-Learning algorithm, with a 20.56% improvement. Of the different neural network algorithms, the DDPG algorithm has the best performance, which has an average path step of 63 steps; compared to the DQN algorithm, it has an increase of 20.25%. When compared to the DDQN algorithm, the number of path steps is increased by 8.69%. According to the above results, the PDQN algorithm is more efficient under the same path conditions, as the learning continues, the fusion algorithm performs better in terms of path steps.

**Figure 9:**
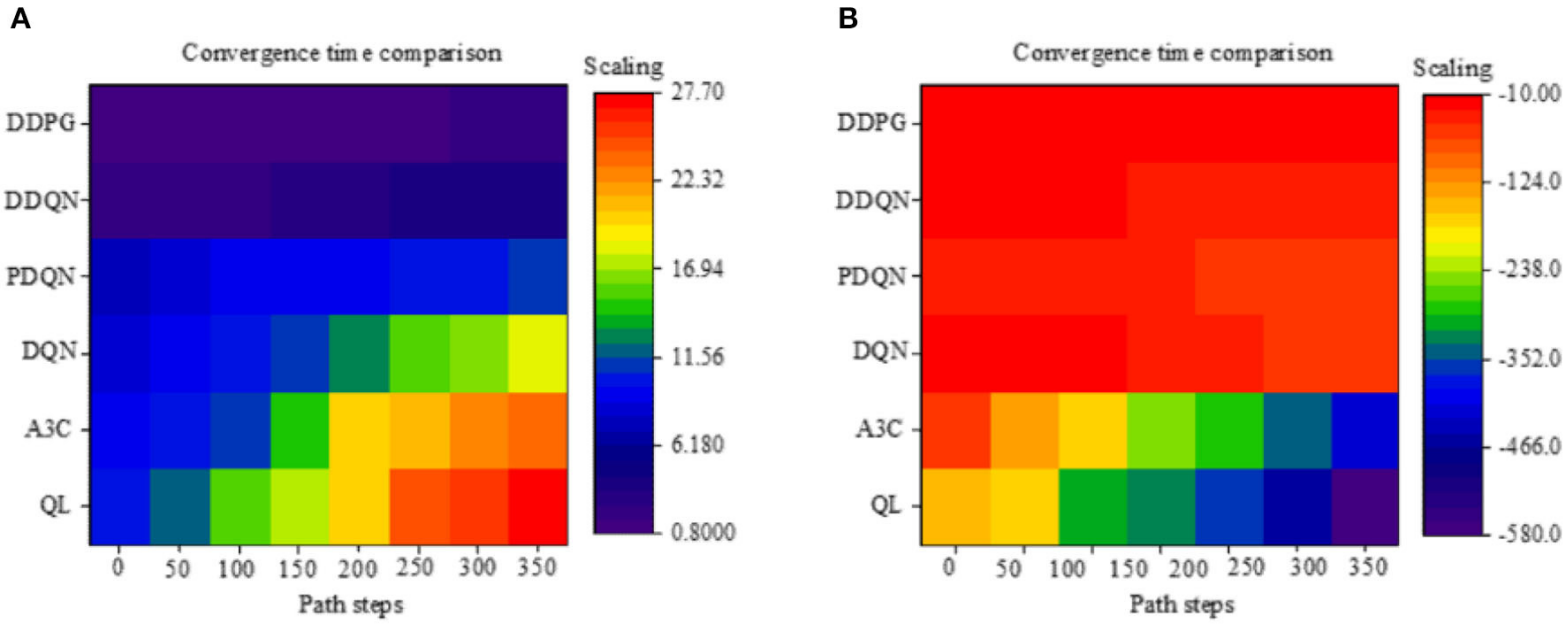
Evaluation of convergence time and cumulative reward performance of different path planning algorithms of the mobile robot (QL algorithm represents the Q-Learning algorithm).

Figure 9A illustrates the convergence time of different algorithms under different path steps. The results show that as the path steps continue to increase, the convergence time of each algorithm is continuously increasing. Compared to the Q-Learning and A3C algorithms, after adding reinforcement learning, the convergence time of robot path planning is increased by 13.54%; compared to the Q-Learning and DQN algorithms, after adding the neural network algorithm, the convergence time of robot path planning is increased by 33.85%, which is the most obvious improvement. Comparing different neural networks, it was found that the convergence time of the DDQN algorithm with increased *Q*-value is greatly improved, and the convergence time of path planning is improved by 94.44% compared with the previous Q-Learning algorithm. For the DDPG algorithm based on neural network and HRL, the convergence time of the algorithm under the unsynchronized number is 1.34 s on average, which is 55.52% faster than the optimal DDQN algorithm.

Figure 9B illustrates the cumulative rewards of different algorithms under different path steps. Since the designed reward rules are more stringent, the reward results are all negative, but this does not affect the obtained results. As shown in Figure 9B, as the number of path steps continues to increase, the cumulative rewards continue to increase. For different algorithms, comparing the Q-Learning and A3C algorithms, the cumulative reward is significantly improved by 29.64%. Compared to the Q-Learning algorithm, the neural network DQN has increased significantly. Under the same neural network, it was found that the PDQN algorithm that introduces the force field has less cumulative rewards. The reason may be that the purpose of the algorithm is to enhance the running speed of the algorithm. The mechanism for rewards is not very complete; thus, the rewards are less. Among the neural network algorithms, the DDQN algorithm has the best cumulative reward. However, compared to the fusion algorithm DDPG, the performance of the DDQN algorithm is not very good. The cumulative reward of DDPG is increased by 41.5% compared to DDQN. According to the above results, it is concluded that under different path steps, the convergence time of the algorithm is the fusion algorithm; at the same time, the algorithm can also obtain the most rewards.

### Analysis of Performance Changes in Neural Network and HRL Algorithms Under Different Environmental Conditions

To explore the impact of different environmental conditions on the performance of the algorithm, the performance of the DDPG algorithm was tested under different action sets, grid numbers, state sets, and force values. Under the premise of the same starting point and ending point, the average value of the algorithm was obtained after running 30 times. The results are shown in Table 1. As shown in the table, the comparison between M1 and M2 indicates that when the action set is doubled, the convergence time of the algorithm will increase by 41%, and the smoothness of the planned path is also increased by 53%. Comparing M2 and M3, it is found that when the number of grids is increased three times, the convergence of the algorithm will be reduced by 69%, and the smoothness will be increased by 45%. Comparing M3 and M4, it was found that increasing the number of state sets will slow down the convergence speed of the algorithm, but by adjusting the direction of the action set, the right angles and corners in the path can be avoided, and the smoothness with which it navigates the planned path is increased by 18%. Comparing M4 and M5, it is found that the introduction of the force field will reduce the convergence time of the algorithm by 49%, which can increase the action step size, thereby adjusting the number of state sets and the direction of the action set. Therefore, when the action set is 4, the number of grids is 3, and the state set is 40^*^40^*^8, with the introduction of the force value, the algorithm can reduce the convergence time by 91% compared with the traditional Q-learning algorithm, and the smoothness of the path increased by 79%.

**Table 1:** Effect of different environmental conditions on algorithm performance.

**Numbering**	**Number of states**	**Number of actions**	**Action step**	**Potential field/s**	**Convergence time**	**Convergence round**	**Path length**	**Total corner/rad**
M1	40*40	4	1	N0	1.9254	682.6	38.1	21.677
M2	40*40	8	1	N0	2.7139	629.7	32.7	10.210
M3	40*40	8	3	N0	0.8515	274.6	34.3	5.655
M4	40*40*8	4	3	N0	1.4259	340.1	32.8	4.616
M5	40*40	4	1	Yes	0.9848	559.8	38.0	21.834
M6	40*40*8	4	3	Yes	0.1735	155.3	32.1	4.555

### Analysis of Changes in Paths Based on Neural Networks and HRL Under Different Scenario Conditions

**Figure 10:**
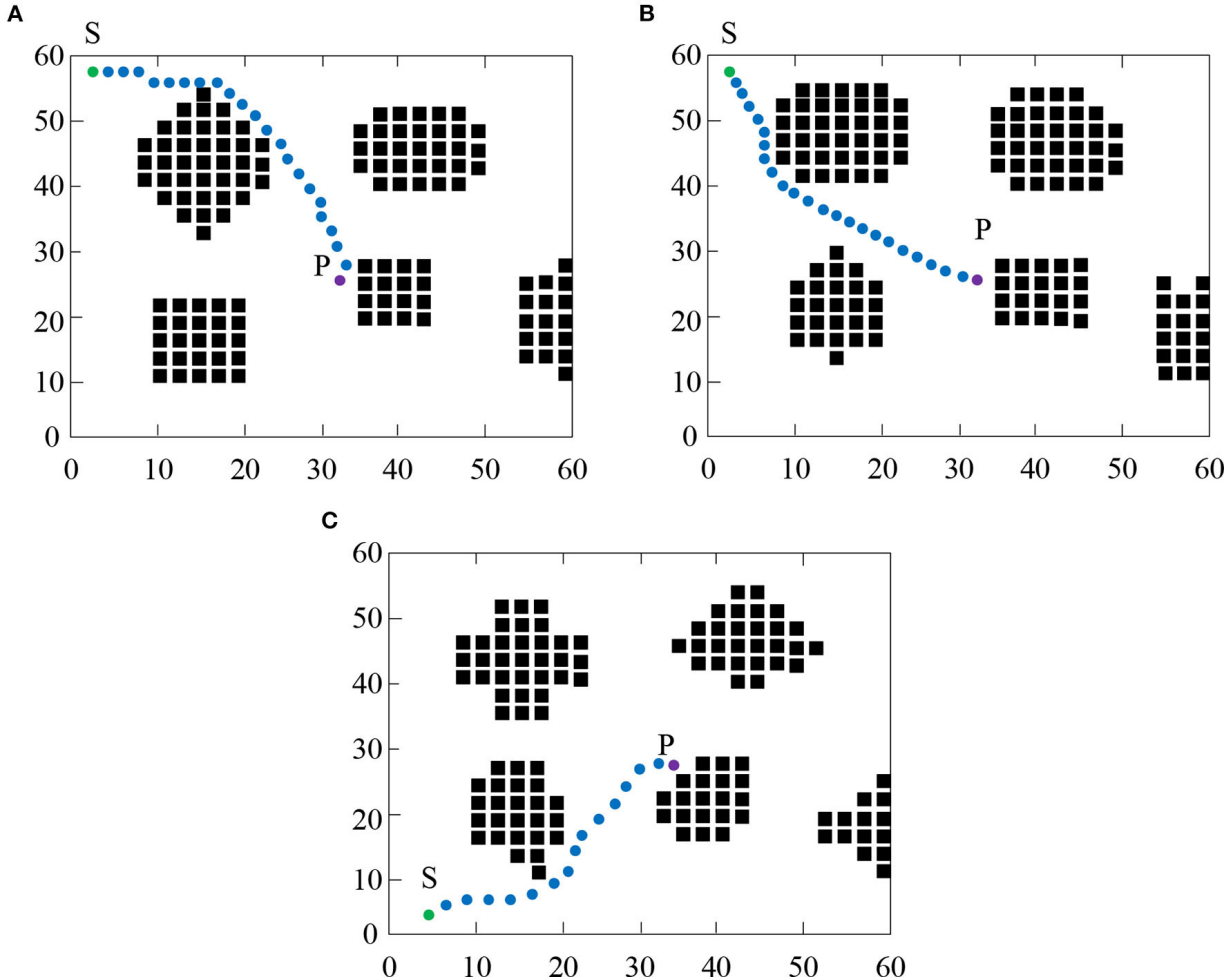
Path changes of algorithms in different scenarios.

Figure 10 and Table 2 indicate the path changes and quantitative data of the algorithm under different scene conditions. As shown in Figure 10, by comparing Figures 10A,B, it was found that at the same starting point and ending point, under the condition of different obstacles, the algorithm system can effectively avoid obstacles and design the optimal paths. In addition, the convergence time is maintained at about 0.15 s, the number of convergence rounds is maintained at 145, and the total rotation angle is 4.8 rad. By comparing Figures 10A,C, it was found that under different environments and different starting points and ending points, the system can still avoid collisions with obstacles, maintain a high convergence time, and design an optimal path. Simulation results show that the proposed path planning algorithm for mobile robots based on neural networks and HRL has a good generalization effect in different scenarios.

**Table 2:** Statistical results of algorithm path changes under different scenario conditions.

**Scenes**	**Position**	**Convergence time/s**	**Convergence round**	**Path length**	**Total corner/rad**
P1	(1, 39)	0.1615	144.5	32.1	4.869
P2	(1, 39)	0.1468	147.0	31.6	4.712
P3	(39, 39)	0.1724	147.4	31.8	4.641

## Discussion

The neural network DQN can perceive the environment and perform feature extraction to realize the fitting from the environment to the state action function. This has been mentioned in the literature. Qiao et al. (2018) proposed an adaptive DQN strategy and applied it to text recognition. These results showed that the DQN algorithm is significantly better than other algorithms, which also indicated the advantages of the DQN algorithm in image recognition (Qiao et al., 2018). Compared with the deep learning algorithm DQN, the DDQN algorithm is better than DQN in terms of value accuracy and strategy, which is also consistent with previous reports (Qu et al., 2020). The hierarchical reinforcement learning technology is utilized to achieve the mapping from state to action and meet the mobile needs of mobile robots. The data have also proven that the robot path planning method based on deep reinforcement learning is an effective end-to-end mobile robot path planning method, which has also been confirmed in a study by Wang B. et al. (2020). The above results illustrate the feasibility of the proposed method in the path planning of mobile robots.

The DDPG algorithm was developed based on the DQN algorithm. The biggest improvement is that the action strategy of the DQN algorithm can only select actions in discrete action space, while the DDPG algorithm can select actions in continuous action space. The results show that the algorithm is significantly better than other algorithms in terms of operating efficiency. This is consistent with the results of Shen X. et al. (2019), in which it was found that when compared with the exponential moving average the effective variance of DDPG and average DDQN were reduced, which explained the efficient runtime of the algorithm further (Shen X. et al., 2019). The results also found that after reinforcement learning is added, the convergence time of robot path planning is increased by 13.54%. Low et al. used the flower pollination algorithm to properly initialize the *Q*-value, which could speed up the convergence of mobile robots (Low et al., 2019). The principle is similar to reinforcement learning, therefore, the research results here are also supported. The comparison between the Q-Learning and DQN algorithms found that the convergence time of robot path planning is increased by 33.85% after adding the neural network algorithm. Some scholars have improved the convergence performance of the model significantly by using two natural heuristic algorithms in unknown or partially known environments (Saraswathi et al., 2018). This natural heuristic algorithm is similar to the neural network structure, further proving the effectiveness of the proposed algorithm.

In summary, the proposed DDQN algorithm has been proven to be applicable to image feature extraction, and the neural network algorithm has also been proven to effectively improve the performance and convergence of the algorithm. The data obtained are consistent with previous research. However, in terms of algorithm performance, the performance of mobile robot path planning based on neural networks and hierarchical reinforcement learning has been significantly improved. This algorithm can significantly reduce path planning time and improve smoothness, enabling mobile robots to move more conveniently and flexibility.

## Conclusions

Through neural networks, the fitting from the environment to the state action function was realized by perceiving the environment and performing feature extraction. Through the enhancement function, the mapping of the current state to the action of the hierarchical reinforcement learning was satisfied, thereby enabling the robot to become more mobile. The two were organically combined to improve the performance of mobile robots during path planning. The mobile robot path planning algorithm based on neural networks and hierarchical reinforcement learning has better performance than other algorithms in all aspects. In addition, the proposed algorithm reduces the planning time, decreases the number of path steps, shortens the convergence time, and increases the smooth and efficient recognition and movement functions of the mobile robots. Although the performance of each algorithm has been analyzed as comprehensively as possible, the following aspects need to be improved in the future. First, it is impossible for the neural network learning method of the mobile robot's motion path planning to perform multiple “trial and error” processes in actual operations, which makes it difficult to apply the proposed algorithm. It is therefore necessary to implement the application on the physical platform before applying the algorithm to the actual robots. Second, the path planning only involves static scenarios. Whether the algorithm can show the same performance when encountering dynamic environmental changes is yet to be explored. The path planning capabilities of mobile robots were improved, laying a theoretical foundation for practical applications.

## Data Availability Statement

The raw data supporting the conclusions of this article will be made available by the authors, without undue reservation.

## Ethics Statement

The studies involving human participants were reviewed and approved by Chongqing University Ethics Committee. The patients/participants provided their written informed consent to participate in this study.

## Author Contributions

All authors listed have made a substantial, direct and intellectual contribution to the work, and approved it for publication.

## Conflict of Interest

The authors declare that the research was conducted in the absence of any commercial or financial relationships that could be construed as a potential conflict of interest.
